# Fabrication of a Polycaprolactone/Alginate Bipartite Hybrid Scaffold for Osteochondral Tissue Using a Three-Dimensional Bioprinting System

**DOI:** 10.3390/polym12102203

**Published:** 2020-09-25

**Authors:** JunJie Yu, SuJeong Lee, Sunkyung Choi, Kee K. Kim, Bokyeong Ryu, C-Yoon Kim, Cho-Rok Jung, Byoung-Hyun Min, Yuan-Zhu Xin, Su A Park, Wandoo Kim, Donghyun Lee, JunHee Lee

**Affiliations:** 1 Department of Nature-Inspired System and Application, Korea Institute of Machinery & Materials, 156 Gajeongbuk-Ro, Yuseong-Gu, Daejeon 34103, Korea; junjie0801@hotmail.com (J.Y.); psa@kimm.re.kr (S.AP.); wdkim@kimm.re.kr (W.K.); 2 Department of Biomedical Engineering, School of Integrative Engineering, Chung-Ang University, 221 Heukseok-Dong, Dongjak-Gu, Seoul 156-756, Korea; 3 Medical Device Convergence Center, Konyang University Hospital, 158 Gwanjedong-Ro, Seo-Gu, Daejeon 35365, Korea; sujeong12@gmail.com; 4 Department of Biochemistry, Chungnam National University, 99 Daehak-ro, Yuseong-gu, Daejeon 34134, Korea; eoslight@cnu.ac.kr (S.C.); kimkk@cnu.ac.kr (K.K.K.); 5 Department of Laboratory Animal Medicine, College of Veterinary Medicine, Seoul National University, 1 Gwanak-ro, Gwanak-gu, Seoul 08826, Korea; hobitmilk@snu.ac.kr; 6 Department of Medicine, School of Medicine, Konkuk University, 120 Neungdong-ro, Gwangjin-gu, Seoul 05029, Korea; vivavets@gmail.com; 7 Korea Research Institute of Bioscience and Biotechnology (KRIBB), 125 Gwahak-ro, Yuseong-Gu, Daejeon 34141, Korea; crjung@kribb.re.kr; 8 Department of Orthopedic Surgery, School of Medicine, Ajou University, 206 World Cup-ro, Yeongtonggu, Suwon 16499, Korea; dr.bhmin@gmail.com; 9 Department of Engineering Mechanics, School of Mechanical and Aerospace Engineering, Jilin University, No. 5988, Renmin Street, Changchun 130025, China; xyz0208@jlu.edu.cn

**Keywords:** progenitor cell, three-dimensional bioprinting system, hybrid scaffold, osteochondral tissue

## Abstract

Osteochondral defects, including damage to both the articular cartilage and the subchondral bone, are challenging to repair. Although many technological advancements have been made in recent years, there are technical difficulties in the engineering of cartilage and bone layers, simultaneously. Moreover, there is a great need for a valuable in vitro platform enabling the assessment of osteochondral tissues to reduce pre-operative risk. Three-dimensional (3D) bioprinting systems may be a promising approach for fabricating human tissues and organs. Here, we aimed to develop a polycaprolactone (PCL)/alginate bipartite hybrid scaffold using a multihead 3D bioprinting system. The hybrid scaffold was composed of PCL, which could improve the mechanical properties of the construct, and alginate, encapsulating progenitor cells that could differentiate into cartilage and bone. To differentiate the bipartite hybrid scaffold into osteochondral tissue, a polydimethylsiloxane coculture system for osteochondral tissue (PCSOT) was designed and developed. Based on evaluation of the biological performance of the novel hybrid scaffold, the PCL/alginate bipartite scaffold was successfully fabricated; importantly, our findings suggest that this PCSOT system may be applicable as an in vitro platform for osteochondral tissue engineering.

## 1. Introduction

Cartilage repair is a limited process. Although many types of cellular therapies are being applied in regenerative medicine, cartilage lesions are difficult to repair. Moreover, when such lesions expand into the subchondral bone, repair processes are even more challenging because both subchondral bone repair and cartilage repair must be stimulated.

To overcome these challenges, three-dimensional (3D) engineered predesigned tissue grafts have been developed. Notably, the use of such matrix scaffolds to mimic the 3D cell environment may contribute to the improvement of tissue engineering for the treatment of osteochondral defects. Indeed, these scaffolds would be expected to provide a biocompatible environment for cell attachment, growth, and differentiation, thereby facilitating tissue formation. Traditional scaffold fabrication technologies include solvent casting/particulate leaching, melting molding, hydrocarbon templating, and freeze drying [1]. However, these methods have some limitations; for example, the architectures are not customized or reproducible, and the fabrication process is harmful owing to solvent toxicity [1]. Furthermore, cell seeding is associated with cell lost and uneven distribution onto the scaffolds [2].

In recent studies, 3D bioprinting technology using biodegradable materials has attracted much interest in tissue engineering. Based on solid freeform fabrication with rapid prototyping, predesigned scaffolds can be fabricated with controllable lines/widths, porosities, and mechanical properties [3]. Various biomaterials are used in the fabrication of scaffolds. The materials can be broadly categorized into synthetic and natural materials. Hydrogels from natural sources are excellent biomaterials, providing an appropriate environment for cells, including good biocompatibility, rich water content, and a natural extracellular matrix (ECM) [4]. Additionally, hydrogels that encapsulate cells, such as alginate, gelatin, and collagen, have been used for fabrication of human tissues using a 3D bioprinting system [5,6,7]. Alginate is suitable and adjustable for tissue engineering in bone, cartilage, and vascular tissue regeneration owing to its biocompatibility, easy handling, and rapid solidification induced by calcium ions [8,9]. However, alginate shows poor mechanical strength [8]. In contrast, polycaprolactone (PCL) is widely used as a biomaterial to enhance the mechanical properties of alginate owing to its superior mechanical strength, low toxicity, and slow degradation rate. Many trials have been performed using both materials in hybrid biomedical scaffolds for tissue engineering applications [2,10,11,12,13,14,15].

The choice of cell sources is also an important determinant of the successful fabrication of an ideal scaffold. Among cell sources, stem cells, chondrocytes, and osteocytes are typically considered good candidates and these efforts for fabrication of osteochondral tissues have been reported [16,17,18]. Fetal cartilage-derived progenitor cells (FCPCs) exhibit high proliferation and multipotency compared with mesenchymal stem cells [19], which could facilitate the fabrication of two heterogeneous tissues in a single construct.

To date, although many advancements in osteochondral strategies using 3D bioprinting systems have been reported, the technical limitations in the differentiation of cells into cartilage and bone in one construct at the same time have not yet been overcome. Moreover, for osteochondral repair, researchers have primarily focused on bonding the cartilage and bone after they are manufactured or obtaining osteochondral scaffolds generated using stem cells seeded onto one scaffold and differentiating them into bone and cartilage [17,20,21]. However, successful differentiation with maintenance of the phenotype of each tissue and difficulties with cultivation have made this technique challenging to apply in practice. Thus, few studies have comprehensively investigated the biological performance of encapsulated FCPCs in hybrid scaffolds.

Accordingly, in this study, we successfully developed a bipartite hybrid scaffold of osteochondral tissue using a lab-made 3D bioprinting system. In addition, to provide the appropriate environments for the differentiation of FCPCs into osteochondral tissue, a polydimethylsiloxane (PDMS) coculture system for osteochondral tissue (PCSOT) was designed and developed. PDMS was used because it is commonly applied as a substrate for cell-based systems [22,23]. Finally, we evaluated osteochondral formation using cell viability, differentiation, biochemical, and histological assays.

## 2. Materials and Methods

### 2.1. Materials

The following materials and reagents were prepared. PCL (average Mn = 45,000), alginic acid sodium salt from brown algae (alginate), calcium chloride (CaCl_2_), sodium citrate, dexamethasone, l-ascorbate acid, sodium pyruvate, l-proline, β-glycerophosphate disodium salt hydrate, sucrose, formalin solution, Alcian blue 8GX, and Alizarin red were purchased from Sigma-Aldrich (St. Louis, MO, USA). PDMS (Sylgard 184) was obtained from Dow Corning (Midland, MI, USA). Phosphate-buffered saline (PBS), fetal bovine serum (FBS), and Dulbecco’s modified Eagle’s medium/high glucose (DMEM/high glucose) were purchased from Hyclone (Logan, UT, USA). Penicillin/streptomycin (P/S; 10,000 U/mL penicillin, 10,000 μg/mL streptomycin), alpha modification of Eagle’s medium (α-MEM), and Insulin-Transferrin-Selenium (ITS; 100×) were obtained from Gibco (Grand Island, NY, USA). The Cell Counting Kit-8 (CCK-8) was purchased from Dojindo (Kumamoto, Japan). The Live/dead kit and Quant-iT PicoGreen dsDNA assay kit were obtained from Thermo Fisher Scientific (Carlsbad, CA, USA). Transforming growth factor (TGF)-β3 and bone morphogenetic protein 2 (BMP-2) were obtained from R&D Systems (Minneapolis, MN, USA) and PeproTech Inc. (Rocky Hill, NJ, USA), respectively. Bovine serum albumin (BSA) was purchased from Merck (Kenilworth, NJ, USA), and the Blyscan assay kit was obtained from Biocolor (Carrickfergus, UK). The Tracp & ALP assay kit and alkaline phosphatase (ALP) were obtained from TaKaRa (Shiga, Japan). The Hybrid-R^TM^ RNA purification kit was purchased from GeneAll (Seoul, Republic of Korea). M-MLV reverse transcriptase was obtained from Promega (Madison, WI, USA). Prime Q-Master mix was purchased from GeNet Bio (Daejeon, Republic of Korea). Optimal cutting temperature (OCT) compound was purchased from Sakura Finetek (Torrance, CA, USA).

### 2.2. Terminology

For clarity, the “hybrid scaffold” refers to the PCL/alginate hybrid scaffold generated in this study, composed of PCL and cell-laden alginate. The “bipartite hybrid scaffold” refers to a structure with the PCL/alginate hybrid scaffold printed onto a PCL membrane in a vertical direction.

### 2.3. 3D Bioprinting System

**Figure 1:**
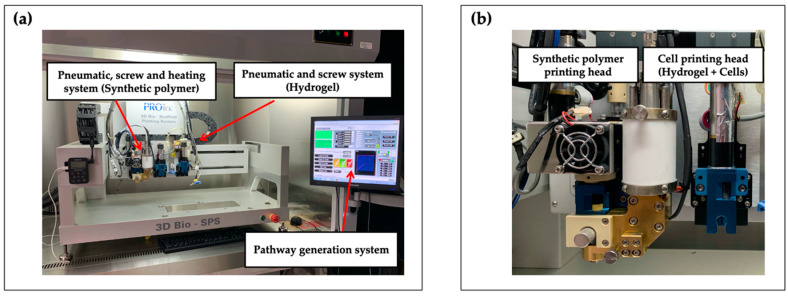
Front view of (**a**) the 3D bioprinting system and of (**b**) its printing heads.

The PCL/alginate hybrid and bipartite hybrid scaffolds were fabricated using a lab-made 3D bioprinting system (Figure 1). The bioprinting system consisted of two or more dispensing heads with multiple types of biomaterials. One was a heating jacket composed of a plunger pneumatic and screw pump system for fabrication of the synthetic polymeric scaffold using high temperature. The other head had a plunger pneumatic and screw pump system that enabled fabrication of the cell-laden hydrogel at room temperature (RT). All dispensing heads could be individually controlled and discretely assembled. To prevent environmental contamination, the 3D bioprinting system was set onto a clean bench.

### 2.4. Progenitor Cell Isolation and Culture

FCPCs were supplied by the Ajou University Medical Center (Suwon, Republic of Korea). The cell isolation procedure was described in a previous study [19]. Human fetal cartilage tissues were obtained from donors undergoing elective termination after obtaining written informed consent. Cells (1 × 10^6^ cells) were seeded onto a 150-mm culture dish and cultured in DMEM containing with 10% FBS and 1% P/S. The culture medium was changed twice a week. FCPCs were passaged at 90% confluence and the passages 5–7 were used for cell printing, and cells were mixed in alginate hydrogel at a density of 5 × 10^6^ cells/mL for fabrication of PCSOT.

### 2.5. Printability and Cell Viability Assay with Different Concentrations of Alginate

**Figure 2:**
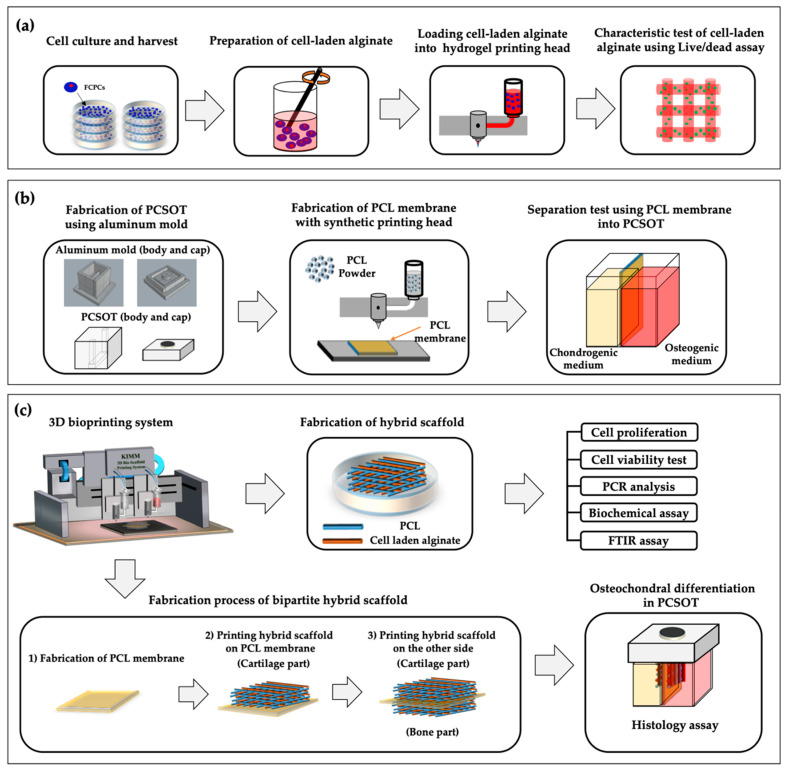
Schematics of the study flow. (**a**) FCPC culture and harvesting for analysis of the characteristics of 1%, 3%, and 5% (w/v) alginate. (**b**) PCSOT design and function test. (**c**) Bioprinted hybrid and bipartite hybrid scaffold; test of the biological performance. Cell-laden alginate was extruded between PCL lines.

In order to analyze whether the printing process and alginate concentration affected the cytocompatibility, printing was performed with 1%, 3%, and 5% (w/v) alginate (Figure 2a). Before encapsulation, alginate powder was sterilized with ethylene oxide (EO) gas. The fabrication process conditions for 1%, 3%, and 5% alginate are introduced below (Table 1).

For the preparation of the alginate solution, the alginate powder was mixed with serum-free DMEM to the final concentration of 1%, 3%, or 5%. The prepared cells were loaded into alginate solution and mixed homogeneously using autoclaved spatulas. To enhance the viscosity of the alginate solution, 1% (w/v) CaCl_2_ was added at a ratio of 17:3 (v/v). After printing the cell-laden structure, 5% (w/v) CaCl_2_ solution in distilled water was crosslinked to maintain the overall shape of the structure. To test the viscosity of the 1%, 3%, and 5% alginate solutions, we used a rheometer (AR 2000 Rheometer; TA Instruments Inc., New Castle, DE, USA).

For observation of the cytocompatibility of alginate under the different concentrations, the structures (n = 4) were stained using a Live/dead kit, according to the manufacturer’s instructions, and incubated for 30 min at room temperature. After incubation, two regions for each image were randomly captured using a confocal microscope (LSM 880; Carl Zeiss, Oberkochen, Germany) to evaluate cell viability after 24 h. Live and dead cells were counted manually using the ImageJ software (NIH, Bethesda, MD, USA). The ratio was calculated as the number of live cells divided by the total number of cells (sum of live and dead cells).

### 2.6. Design and Fabrication of the PCSOT

To provide an appropriate culture environment for the two different types of tissues to mature, a PCSOT was designed and developed. The deigned mold for fabrication of the PCSOT included a body and cap (Figure 2b). To facilitate curing and biocompatibility, the mold was made of aluminum with Teflon coating.

The rigidity of PDMS could be controlled by altering the concentration of the curing agent. Before pouring the PDMS solution into the Teflon-coated aluminum mold, the base and curing agent were mixed evenly at a ratio of 10:1 (v/v). The Teflon coating mold was then baked at a constant temperature of 100 °C for 1 h. To test the separating function of the fabricated PCSOT, the PCL membrane was fabricated (Figure 2b). Solutions with two different colors were then poured into each chamber. Observations were carried out for 28 days.

### 2.7. Fabrication Process of Hybrid and Bipartite Scaffold

After optimization, hybrid scaffolds were fabricated using the bioprinting system. The most important difference between the hybrid scaffold and bipartite hybrid scaffold was that one was printed on a cell culture dish, whereas the other was printed on a PCL membrane in a vertical manner (Figure 2c). Briefly, for the hybrid scaffold, PCL was printed onto cell culture dishes, which were used as a framework to prevent the alginate structure from collapsing. After PCL lines were printed, the alginate solution was added between the PCL lines. Subsequent layers were printed at an angle of 90°, and the process was repeated several times, alternating between the two printing heads. For the evaluation of the osteochondral properties of the samples, we performed CCK-8, Live/Dead, polymerase chain reaction (PCR), biochemical, and Fourier-transform infrared spectroscopic (FT-IR; VERTEX 80v; Bruker, Billerica, MA, USA) assays.

Regarding the bipartite hybrid scaffold, a PCL membrane was fabricated and was used to distinguish chondral and osteogenic tissues. The thickness of the PCL membrane was controlled at 0.6 mm. After printing the PCL membrane, the two hybrid scaffolds were printed on the PCL membrane in a vertical manner. Bipartite scaffolds were observed to determine whether osteochondral tissue differentiated successfully in the PCSOT using Alcian blue and Alizarin red staining.

All scaffolds were cross-linked with 5% (w/v) CaCl_2_ for 10 min to maintain the shape of the whole structure. In addition, all scaffolds were cultured in cell proliferation medium for 1 day to ensure stabilization. Subsequently, the scaffolds were treated with specific induction medium (cell density of all samples: 5 × 10^6^ cells/mL).

### 2.8. Specific Induction Medium Conditions for the Hybrid Scaffold

To analyze the potential for osteochondral differentiation, FCPCs encapsulated in hybrid scaffolds were cultured in chondrogenic or osteogenic medium. For chondrogenic differentiation, the scaffolds were cultured with proliferation medium for 1 day. The medium was then changed to a chondrogenic differentiation medium consisting of DMEM with 1% P/S, 1.25 mg/mL BSA, 100 nM dexamethasone, 50 µg/mL ascorbate-2 phosphate, 100 µg/mL sodium pyruvate, 40 µg/mL l-proline, 10 ng/mL TGF-β3, and 1% ITS (100×). For osteogenic differentiation, cells were incubated with osteogenic differentiation medium containing α-MEM, 10% FBS, 1% P/S, 10 mM β-glycerophosphate disodium salt hydrate, 100 nM dexamethasone, 50 μg/mL ascorbate-2 phosphate, and 100 ng/mL BMP-2. As abovementioned, to provide a better cell-culture environment and improve the biological performance, TGF-β3 and BMP-2 were added to the experimental groups. On the other hand, cultures without the differentiation stimuli were evaluated using the same method, as controls.

### 2.9. Proliferation Assay

FCPCs proliferation in the context of osteogenesis and chondrogenesis was analyzed using the CCK-8 assay at 1, 14, and 28 days. 3D constructs were immersed in 400 μL serum-free DMEM, and 40 μL CCK-8 reagent was added to 48-well plates. Serum-free DMEM without cells was evaluated as the blank control. After 4 h, 100 μL of the solution was transferred to a 96-well plate, and the absorbance at 450 nm was measured using a microplate reader (Model 680; Bio-Rad Laboratories, Hercules, CA, USA). Finally, absorbance values were corrected by subtracting the measurement for the 100-μL blank. Six specimens were prepared for each group.

### 2.10. Cell Viability Assay

To evaluate the cell viability in the composite scaffolds, FCPCs in hydrogels were stained using a Live/dead assay kit and incubated for 30 min in an atmosphere containing 5% CO_2_ at 37 °C. After incubation, images were captured using a fluorescence microscope (Eclipse Ti; Nikon, Tokyo, Japan). Cell viability was examined on days 1 and 28.

### 2.11. RNA Isolation and Quantitative Reverse Transcription PCR (RT-qPCR)

After 14 or 28 days of culture, the hybrid scaffolds (n = 3 per group) were dissolved in 55 mM sodium citrate to decompose the crosslinked alginate [24]. Total RNA was isolated using a Hybrid-R^TM^ RNA purification kit. Reverse transcription was carried out using the M-MLV reverse transcriptase with random hexamers. mRNA levels were measured by qPCR with gene-specific primers (Table 2) using the Prime Q-Master mix on the AriaMx PCR System (Agilent, Santa Clara, CA, USA). All reactions occurred under identical cycling conditions, as follows: 40 cycles of amplification with denaturation at 95 °C for 20 s, annealing at 58 °C for 20 s, and elongation at 72 °C for 20 s. Gel electrophoresis and melting curve analysis was used to verify the specificity of the products amplified by each primer set.

### 2.12. Biochemical and FT-IR Measurement

Glycosaminoglycan (GAG) contents, ALP activity, and the presence of PO_4_^3−^ groups were analyzed to assess the differentiation properties of FCPCs in the hybrid scaffolds. Before biochemical assays, all samples were dissolved in 55 mM sodium citrate to decompose alginate. GAGs are the major component of the cartilage extracellular matrix. Therefore, samples (n = 6) were digested with 1 mL papain solution overnight at 65 °C. After incubation, sulfated GAGs in samples were evaluated using the commercial Blyscan assay kit following the manufacturer’s protocol. The absorbance of sulfated GAGs was measured at 655 nm. For the evaluation of DNA contents, we used a Quant-iT PicoGreen dsDNA kit. A 10-μL aliquot of the lysate with working regent was measured at excitation/emission wavelengths of 485/528 nm using a microplate multi-reader (Spectramax iD3; Molecular Devices, San Jose, CA, USA).

ALP is a membrane-bound glycoprotein that enhances osteogenesis and acts as an early marker of osteogenesis. For the assessment of ALP activity, scaffolds on days 1, 14, and 28 were analyzed using a TRACP & ALP Assay Kit. All scaffolds (n = 6) were washed one time with PBS and one time with physiological saline. After washing, samples were lysed with 150 μL lysis buffer. Next, 100 μL supernatant from lysates was mixed with the same volume of p-nitrophenyl phosphate and was incubated at 37 °C for 40 min. Subsequently, 100 μL supernatant was added to a 96-well plate containing 50 μL of 0.5 N NaOH for stopping the reaction. The ALP standard was generated using calf intestine ALP. The ALP activities of the standard and samples were determined by measuring the absorbance at 405 nm using a microplate multi-reader.

The total protein contents in cell lysates were determined using the Bio-Rad Protein Assay kit (Bio-Rad), according to the manufacturer’s instructions. Briefly, an aliquot of the lysate (diluted with distilled water; total test volume: 10 μL) was added to 200 μL working regent, and the absorbance was measured at 595 nm. Data for ALP activity were expressed as the ratio of ALP per protein (units: μU/μg).

For the observation of PO_4_^3−^ groups in osteogenic samples, samples were washed twice with PBS and then freeze-dried for 24 h. The FT-IR spectrometry was performed to detect BMP-2-induced samples on days 1 and 28.

### 2.13. Histological Assays

The constructs were fixed in 10% formalin, dehydrated using 30% (w/v) sucrose solution, embedded in OCT compound, and then cut into 100-μm-thick sections using a freezing CM1950 microtome (Leica, Wetzlar, Germany). Next, samples were stained with Alcian blue and Alizarin red staining. To detect the secretion of proteoglycans and calcium deposition from osteochondral tissue, samples were stained according to a protocol reported by previous study [16]. In brief, chondrogenesis samples were stained with 1% Alcian Blue 8GX in 3% acetic acid (pH 2.5) for 20 min at room temperature. In order to prevent nonspecific staining, samples were washed several times with PBS. On the other hand, osteogenesis samples were stained with Alizarin Red solution (pH 4.3) for 20 min at room temperature, and then washed with PBS. All stained samples were observed using an optical microscope (Eclipse Ti; Nikon).

### 2.14. Statics Analysis

All results are expressed as the means ± standard deviation. Statistical analysis was performed using the two-way ANOVA with Tukey’s or Sidak’s multiple comparison tests. The Prism version 7 software (GraphPad Software, San Diego, CA, USA) was used for all analyses. Differences with *p* values lower than 0.05 were considered significant.

## 3. Results and Discussion

### 3.1. Fabrication of PCSOT and Separating Function Test

**Figure 3:**
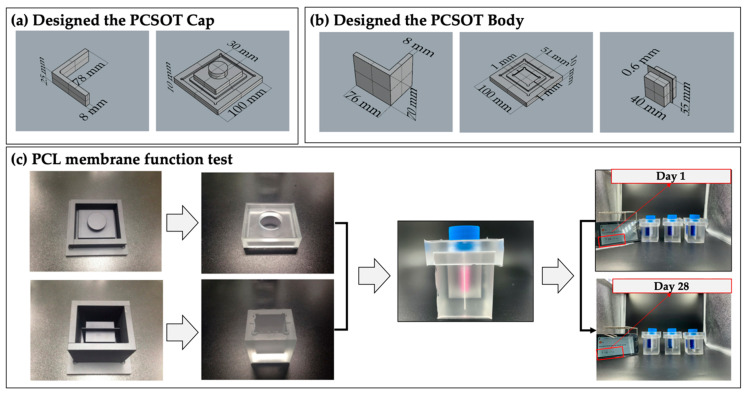
The PCSOT system, the PCL membrane, and the separating function test. (**a**) 3D view of the PCSOT cap in Rhino; (**b**) 3D view of the PCSOT body in Rhino; (**c**) PCL membrane function tests on days 1 and 28.

For the formation and characterization of osteochondral tissues, we designed a PCSOT. Before fabrication, an aluminum mold coated with Teflon was prepared. The aluminum mold was composed of a cap and body. As shown in Figure 3a,b, the cap and body of the mold were modularized and could be easily disassembled. After pouring the PDMS solution into the aluminum mold, the PCSOT was baked at 100 °C in an oven to solidify. Of note, since PDMS is flexible and stable under high temperatures [25], autoclave sterilization can be employed before the cultivation of osteochondral tissues in the PCSOT, which was the case in this study.

As shown in Figure 3c, the PMDS-based PCSOT platform (white ones—silicon) was fabricated using the aluminum-Teflon coated mold (black ones). To prevent contamination from the outside, the cap of a T75 flask was used to close off the PCSOT. The body of the PCSOT consisted of two separated chambers, allowing the cells to be exposed to different culture media. To test whether the PCL membrane fully contained the different culture media, the bipartite hybrid scaffold was fabricated. Over the 28 days of observation, we found no permeation of medium between the two chambers. These results indicated that the PCSOT could completely separate the chambers, providing suitable environments for osteochondral differentiation. Moreover, comparing with other cultivation systems [17,20], the PCSOT system was economical and easy to handle.

### 3.2. Fabrication of the PCL/Alginate Bipartite Scaffold

**Figure 4:**
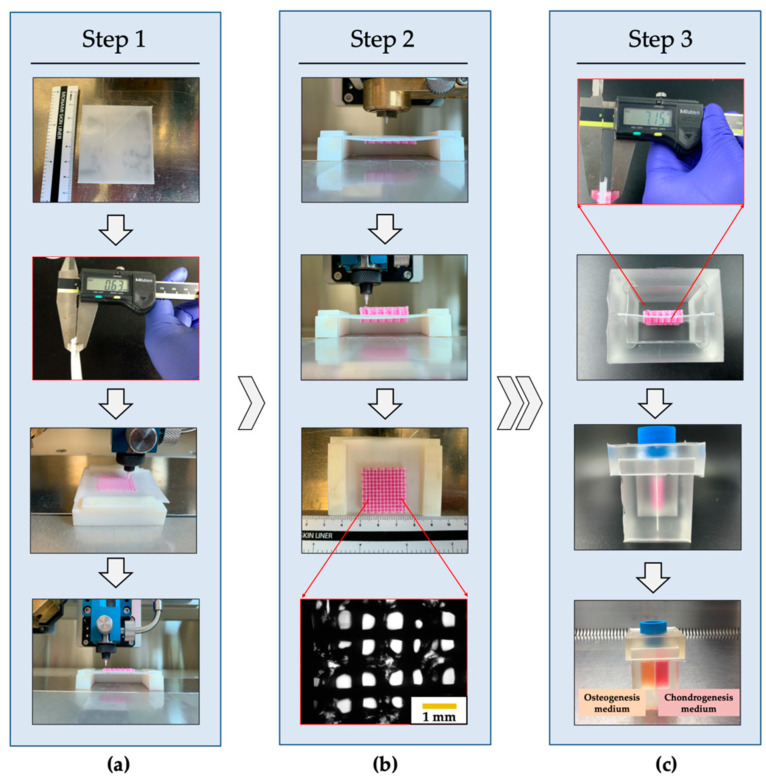
Printing process of the bipartite hybrid scaffold. (**a**) Hybrid scaffold printed on the PCL membrane. (**b**) Hybrid scaffold printed on the other side. (**c**) Bioprinted bipartite hybrid scaffold matured into osteochondral tissue in the PCSOT.

A bipartite hybrid scaffold was successfully fabricated using a 3D bioprinting system (Figure 4). The PCL membrane was used as a partition wall in the PCSOT, and the thickness was controlled at 0.6 mm. Cells were encapsulated in alginate and deposited between preprinted PCL strands. The PCL strands could reinforce the relatively low mechanical properties of alginate as a framework. After one hybrid scaffold was printed on the PCL membrane (Figure 4, step 1), another hybrid scaffold was printed on the other side (Figure 4, step 2). Next, the fabricated bipartite hybrid scaffold was inserted into the PCSOT for observation of osteochondral formation (Figure 4, step 3). Through the microscope, the alginate located between the PCL lines and micro-interconnective pores was observed. Of note, the microporous structure could support encapsulated cells, allowing the exchange of oxygen, nutrients, and waste [14]. The fabrication conditions of the bipartite hybrid scaffold are shown in Table 3 and the size of bipartite hybrid scaffold was 24 × 24 × 7 mm^3^. Each hybrid scaffold was stacked in 12 layers. In this study, comparing with the conventional methods reported [26,27,28], a sophisticated scaffold could be printed in an arbitrary shape by computer-aided design (CAD) software. Moreover, the fabrication process was safe, since the use of solvents was not required. Comparing with the existing studies [20,21], the 3D bioprinting system allowed a one-step fabrication process. In addition, there is no risk of separation due to insufficient adhesion between the two layers.

### 3.3. Evaluation of the Extrusion Performance and Cell Viabiltiy

**Figure 5:**
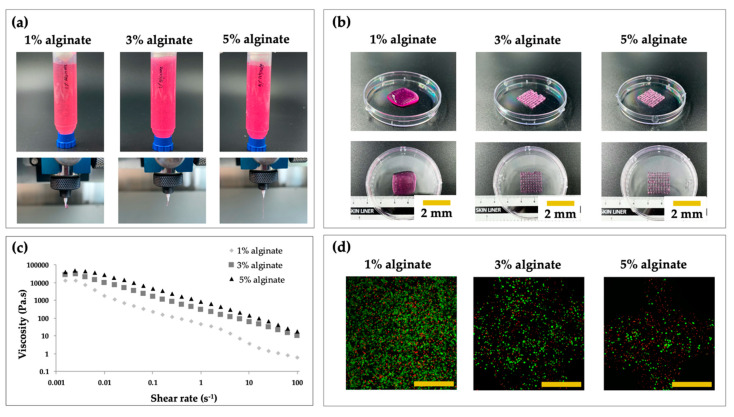
Evaluation of the printability and cell viability of different concentrations of alginate. (**a**) Images of alginate extrusion at 1%, 3%, and 5% (w/v). (**b**) Representative images of cubes printed with different concentrations of alginate. (**c**) Viscosity of samples with different concentrations of alginate. (**d**) Live/Dead confocal microscopy images (scale bar: 500 μm).

Cells encapsulated in the alginate scaffold (1%, 3%, and 5% [w/v] alginate) were fabricated to observe the effects of different weight fractions on cell viability. As shown in Figure 5a, the extruded shapes of the alginate through the nozzle were significantly influenced by the concentration of alginate. In particular, samples with 3% and 5% alginate showed greater uniformity and good stability after printing. Moreover, as shown in Figure 5b, the fabricated structure using 1% alginate showed a liquid-like structure. In contrast, 3D bioprinted scaffolds with 3% and 5% alginate showed greater accuracy than that with 1% alginate. Thus, the results indicated that sufficient viscosity made the printing process easier.

As shown in Figure 5c, the sample prepared with 5% alginate exhibited the highest viscosity among the test groups, indicating that the viscosity was increased as the weight fraction of alginate increased. Moreover, cell viability was significantly decreased as the weight fraction of alginate increased (Figure 5d). As shown in confocal images, a few dead cells were observed; this could have been a result of the shear stress through the micro-sized nozzle, the pneumatic process, or the CaCl_2_ crosslinker [10,14]. The results implied that cell viability was significantly influenced by the concentration of alginate. Cell viability in samples with 3% alginate was approximately 98% relative to that in the context of 1% alginate. However, the cell viability in the context of 5% alginate was significantly lower: approximately 55% relative to that in the context of 1% alginate. Although the higher alginate concentration enabled the structure to be sustained for a long time, cell viability was dramatically decreased. Consequently, based on the results of printability and viability tests, 3% alginate was finally selected.

### 3.4. Analysis of Biocompatibility

**Figure 6:**
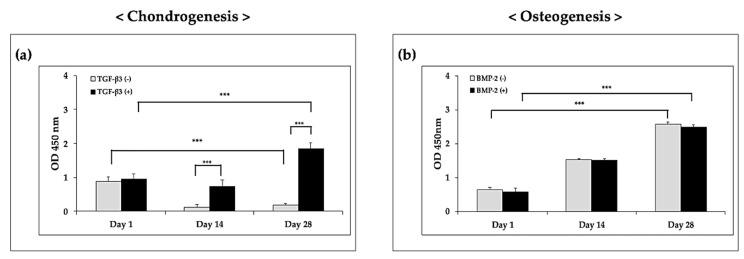
Proliferation results for (**a**) chondrogenesis and (**b**) osteogenesis on the hybrid scaffolds, as per the CCK-8 assays (OD = optical density). *** *p* < 0.001, two-way ANOVA with Tukey’s multiple comparison test.

**Figure 7:**
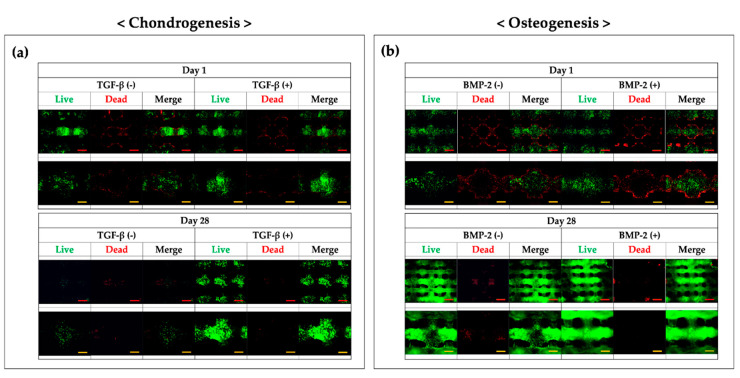
Viability of cells on the hybrid scaffold, as determined using the Live/dead assay. The green and red dots represent live and dead cells, respectively. (**a**) Hybrid scaffolds in chondrogenic induced medium. (**b**) Hybrid scaffolds in osteogenic induced medium. Red bar scale: 1000 μm; yellow bar scale: 500 μm.

To ensure the biocompatibility of the hybrid scaffolds, CCK-8 and Live/dead assays were performed (Figure 6 and Figure 7). The optical density (OD) of the hybrid scaffold in chondrogenic medium supplemented with growth factors was significantly higher than that in the control group on days 14 and 28 (Figure 6a). According to these results, TGF-β3 had positive effects on cell proliferation. Moreover, under osteogenic conditions, although the OD values were significantly increased over time, the addition of BMP-2 did not affect the OD values compared with those in the control group (Figure 6b). These results demonstrated that BMP-2 did not have positive effects on the proliferation of FCPCs.

As shown in Figure 7, live cells were stained as small green dots, and dead cells appeared as red dots. On the printed hybrid scaffolds, cells in alginate were found to be distributed homogeneously on day 1. Although a few dead cells were observed on day 1, most cells survived and proliferated over the long culture period. Interestingly, in the chondrogenic group, the number of live cells increased over time in the presence of TGF-β3. On day 28, many live cells were found in the experimental group, whereas few live cells were observed in the TGF-β3-free group. Similar to the results in Figure 6a, these findings indicated that TGF-β3 had positive effects on cell survival and proliferation. In the osteogenic group, sufficient live cells were observed in the experiment group on day 28. However, there were no significant differences compared with the control group. Overall, these findings suggested that the fabricated microporous and mechanically stable structure provided an appropriate environment for FCPC culture when a suitable medium was used.

### 3.5. Ostechondral Gene Expression and Biochemical Assays Using the Hybrid Scaffolds

**Figure 8:**
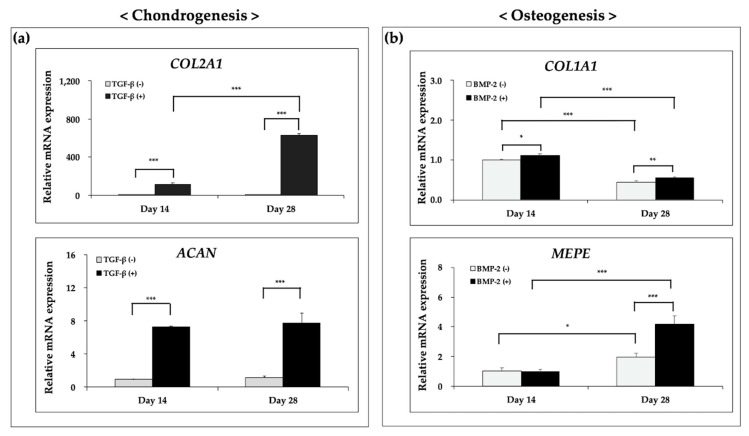
Expression of cartilage- and bone-specific genes. (**a**) Relative expression levels of *COL2A1* and *ACAN;* two-way ANOVA with Sidak’s multiple comparison test. (**b**) Relative expression levels of *COL1A1* and *MEPE*; two-way ANOVA with Tukey’s multiple comparison test. * *p* < 0.05, ** *p* < 0.01, and *** *p* < 0.001.

Chondrogenic and osteogenic genes were analyzed using RT-qPCR. For chondrogenesis, *COL2A1* and *ACAN* were detected (Figure 8a). The mRNA levels of *COL2A1* and *ACAN* were significantly increased compared with those in the control group at both time points analyzed. Notably, the addition of TGF-β3 significantly enhanced chondrogenic formation. In the osteogenic group, *COL1A1* and *MEPE*, which have roles in hard tissue formation, were evaluated. As shown in Figure 8b, the mRNA expression of *COL1A1*, an early marker of bone formation [29], was significantly decreased in the BMP-2-treated group, in agreement with previous studies [30,31]. In contrast, the mRNA levels of *MEPE*, usually upregulated during the maturation of osteoblasts into osteocytes [32], was increased in the BMP-2-treated group. Taken together, these results demonstrated that the growth factors could enhance the differentiation of FCPCs.

**Figure 9:**
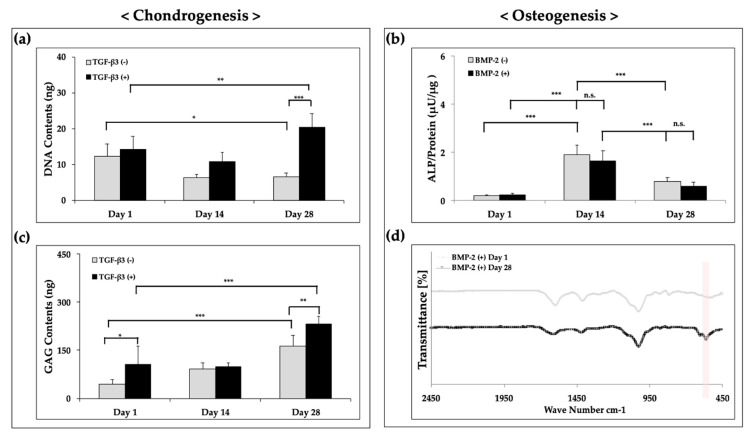
Biochemical and FT-IR measurements results for (**a**) DNA contents, (**b**) ALP activity, (**c**) GAG contents, and (**d**) FT-IR. * *p* < 0.05, ** *p* < 0.01, and *** *p* < 0.001, two-way ANOVA with Tukey’s multiple comparison test in (**a**) and (**b**), two-way ANOVA with Sidak’s multiple comparison test in (**c**).

To evaluate differentiation at the protein level, the scaffolds were used for biochemical assays on days 1, 14, and 28. The DNA and GAG contents in the chondrogenic medium with growth factors were significantly higher than those in the control conditions at each time point (Figure 9a,c). The results showed that TGF-β3 enhanced the ECM production and DNA content in the hybrid scaffold compared with that in the growth factor-free group, similar to the results of cell proliferation in the context of chondrogenesis (Figure 6a). Generally, cell proliferation would be suppressed when cell differentiation occurs [33]. However, some previous studies showed that TGF-β3 had positive effects on both cell viability and differentiation [34,35,36]. Our findings suggested that the addition of growth factors had positive effects on chondrogenesis relative to that in the growth factor-free group. On the other hand, under osteogenic conditions, the scaffolds showed moderate ALP activity (Figure 9b); however, there were no significant differences compared with the control group.

Next, we analyzed the functional characteristics using FT-IR with detection of PO_4_^3−^ forms. A previous study showed that PO_4_^3−^ groups could be detected as bands at 560–600 cm^−1^ and 1000–1100 cm^−1^ [37]. Indeed, as shown in Figure 9d, the peak value was detected as an absorption band at 560–600 cm^−1^, and a significant difference was observed between days 1 and 28. Thus, these results indicated that the hydroxyapatite formed might be driven by BMP-2 stimulation over the 28 days of culture. Taken together, these findings demonstrated that FCPCs in the hybrid scaffolds could undergo chondrogenic and osteogenic differentiation in complete medium with added growth factors.

### 3.6. Histological Findings of Bipartite Hybrid Scaffolds in the PCSOT

**Figure 10:**
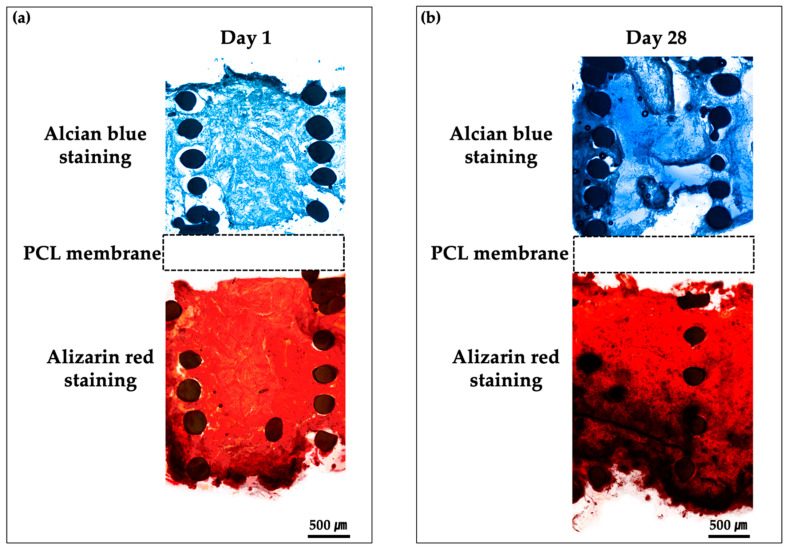
Histological analysis of osteochondral tissue formation using Alcian blue and Alizarin red staining. Samples evaluated at (**a**) day 1 and (**b**) day 28. The bipartite hybrid scaffolds were cultured in the PCSOT for 4 weeks.

To evaluate the differentiation of cells cultured on bipartite hybrid scaffolds in the PCSOT, we performed Alcian blue and Alizarin red staining to assess chondrogenic and osteogenic functional characteristics, respectively (Figure 10). Alcian blue staining was used to detect the sulfated proteoglycans in the cartilage. As shown in Figure 10b, chondrogenic scaffolds cultured in the chondro-induced chamber were strongly stained with Alcian blue compared with day 1. These results indicated that GAGs accumulated over time in this group. In the osteo-induced chambers, the osseous phase was positively stained with Alizarin red versus day 1. Darker red colors were observed as calcium phosphate minerals were deposited on day 28. These results showed that the PCSOT completely separated the specific differentiation media; therefore, the engineered PCL/alginate bipartite scaffold could be successful differentiated into osteochondral tissues in our platform.

## 4. Conclusions

Osteochondral tissue regeneration is an interesting challenge in tissue engineering. To overcoming this challenge, a 3D bioprinting system was used to simultaneously print two independent tissues for osteochondral tissue formation. The hybrid scaffolds were fabricated and their biological performance was evaluated under osteochondral differentiation conditions. Accordingly, our results showed that the addition of growth factors led to a better stimulation of osteochondrogenesis compared with that in the context of growth factor free groups. In addition, an in vitro platform of PCSOT was successfully developed with the aim of establishing a system for the repair of osteochondral defects. In this study, we identified the building potential of PCL/alginate bipartite hybrid scaffolds using a 3D bioprinting system with progenitor cells. Moreover, the PCSOT was successfully employed using distinct chondrogenic and osteogenic media, allowing FCPCs to undergo differentiation into osteochondral tissues. Future studies are needed to assess the application of 3D hybrid scaffolds with the PCSOT for uses in the context of engineering multiphase tissue formation.

## Figures and Tables

**Table 1:** Fabrication processes for the conditions of 1%, 3%, and 5% alginate.

Material	Pressure (kPa)	Temperature (°C)	Speed (mm/min)	Screw (rpm)	Nozzle (µm)
1% Alginate	60	RT	440	39	300
3% Alginate	90	RT	440	39	300
5% Alginate	125	RT	440	39	300

**Table 2:** RT-qPCR primers utilized for detection of osteogenic and chondrogenic gene expression.

Gene		Direction (5′-3′)	Primer Sequence
Cartilage-specific genes	*COL2A1*	Forward	GTTCACGTACACTGCCCTGA
		Reverse	TCCACACCGAATTCCTGCTC
	*ACAN*	Forward	CCTCTGCATTCCACGAAGCTAAC
		Reverse	TGCCTCTGTCCCCACATCAC
Bone-specific genes	*COL1A1*	Forward	GTGTTCCTGGAGACCTTGGC
		Reverse	CACCAGCATCACCCTTAGCA
	*MEPE*	Forward	TGCGAGTTTTCTGTGTGGGAC
		Reverse	TCTTCCACACAGCTTTGCTTAG
Reference gene	*GADPH*	Forward	TTGAGGTCAATGAAGGGGTC
		Reverse	GAAGGTGAAGGTCGGAGTCA

**Table 3:** Fabrication conditions of the hybrid and bipartite hybrid scaffolds.

Material	Pressure (kPa)	Temperature (°C)	Speed (mm/min)	Screw (rpm)	Nozzle (μm)
PCL strand	290	110	440	52	300
Alginate strand	90	RT	440	39	300
PCL membrane	290	110	800	52	400
